# Reproducibility of a Questionnaire for Dietary Habits, Lifestyle and Nutrition Knowledge Assessment (KomPAN) in Polish Adolescents and Adults

**DOI:** 10.3390/nu10121845

**Published:** 2018-12-01

**Authors:** Joanna Kowalkowska, Lidia Wadolowska, Jolanta Czarnocinska, Magdalena Czlapka-Matyasik, Grzegorz Galinski, Marzena Jezewska-Zychowicz, Monika Bronkowska, Anna Dlugosz, Dorota Loboda, Joanna Wyka

**Affiliations:** 1 Department of Human Nutrition, University of Warmia and Mazury in Olsztyn, Słoneczna 45F, 10-718 Olsztyn, Poland; lidia.wadolowska@uwm.edu.pl; 2 Behavioral Conditions of Nutrition Team, Committee of Human Nutrition Science, Polish Academy of Sciences, 00-901 Warsaw, Poland; jolanta.czarnocinska@up.poznan.pl (J.C.); marzena_jezewska_zychowicz@sggw.pl (M.J.-Z.); 3 Institute of Human Nutrition and Dietetics, Poznan University of Life Sciences, Wojska Polskiego 28, 60-637 Poznan, Poland; magdam@up.poznan.pl (M.C.-M.); gregoryg@up.poznan.pl (G.G.); 4 Department of Organization and Consumption Economics, Warsaw University of Life Sciences-SGGW, Nowoursynowska 159 C, 02-776 Warsaw, Poland; 5 Department of Human Nutrition, Wrocław University of Environmental and Life Sciences, Chełmońskiego 37, 51-630 Wroclaw, Poland; monika.bronkowska@upwr.edu.pl (M.B.); joanna.wyka@upwr.edu.pl (J.W.); 6 Faculty of Chemical Technology and Engineering, University of Technology and Life Sciences in Bydgoszcz, Seminaryjna 3, 85-326 Bydgoszcz, Poland; ad.dlugosz@gmail.com; 7 Institute of Health, University of Economy in Bydgoszcz, Garbary 2, 85-229 Bydgoszcz, Poland; dorota@byd.pl

**Keywords:** FFQ, eating behaviours, diet quality index, nutrition knowledge, physical activity, reliability, internal consistency, kappa coefficient

## Abstract

The aim of the study was to evaluate the reproducibility of the Dietary Habits and Nutrition Beliefs Questionnaire (KomPAN) in Polish adolescents and adults, including the assessment of indexes developed based on the questionnaire. In total, the study involved 954 subjects aged 15–65 (53.9% females). Interviews using the interviewer-administered questionnaire (IA-Q) in healthy subjects (*n* 299) and the self-administered questionnaire (SA-Q) in healthy subjects (*n* 517) and outpatients (*n* 138) were conducted and repeated after two weeks. Considering the consumption frequency of 33 food items, the cross-classification (test-retest) agreement of classification into the same category obtained for IA-Q in healthy subjects ranged from 72.2% (fruit juices) to 91.6% (energy drinks); the kappa statistic was >0.60 for all food items. For SA-Q conducted in healthy subjects the cross-classification agreement ranged from 63.8% (vegetable oils, margarines, mixes of butter and margarines) to 84.7% (lard); the kappa statistic was >0.50 for all food items. For SA-Q in outpatients, the cross-classification agreement ranged from 42.0% (both fruit juices and white rice, white pasta, fine-ground groats) to 92.0% (energy drinks); the kappa statistic was ≥0.40 for 20/33 food items. The kappa statistic for lifestyle items ranged 0.42–0.96, and for the nutrition knowledge level it ranged 0.46–0.73. The questionnaire showed moderate to very good reproducibility and can be recommended to assess dietary habits, lifestyle and nutrition knowledge of healthy adolescents and adults and those suffering from chronic diseases, after validation and/or calibration study is carried out. The reproducibility of the interviewer-administered questionnaire was better than its self-administered version. The reproducibility of the self-administered questionnaire was better in healthy subjects than in outpatients.

## 1. Introduction

Food frequency questionnaires (FFQs) are commonly used due to their low cost, quick and easy administration [1,2,3]. FFQs evaluate the habitual diet over a long period of time (e.g., a year) allowing the ranking of respondents into categories of habitual consumption of foods, specific food components or nutrients as well as identifying dietary patterns [2,4]. Based on FFQs, various diet quality scores were developed as brief tools to assess compliance with dietary recommendations [5,6,7]. In comparison with other nutrition assessment methods, the advantages of FFQs predispose their application in large epidemiological studies to assess diet-disease relationship [3,4]. In many countries, FFQs were developed (or adapted) and used in healthy subjects [8,9,10,11,12] as well as in relation to various health outcomes, such as obesity, cardiovascular disease, diabetes or cancer [13,14]. However, the application of culture- or country-specific tools is preferred and highly recommended, due to differences in commonly consumed foods, traditional dishes or serving sizes [1,8]. 

Each newly-created tool should be validated in the population that it was designed for [1,4]. A range of random and systematic errors may reduce the validity of a questionnaire [4]. To determine whether a food-frequency questionnaire provides reproducible results is important for all applications of FFQs [1]. Furthermore, the assessment of reproducibility is necessary to apply a newly developed or adopted questionnaire in future research [1,9]. The FFQs can be developed in both versions–an interviewer-administered and a self-administered [4], although their reproducibility can differ [1,15]. According to Cade et al. [1], two-thirds of the questionnaires in validation studies were self-administered. An assessment of the effect of mode of questionnaire administration is crucial for the methodology of future studies using the questionnaire and the validity of the findings [15]. 

Developing a more comprehensive questionnaire containing questions not only about the frequency of food consumption, but also on nutrition knowledge and other dietary and lifestyle behaviours, can be essential for a complex assessment of multidimensional dependencies between habitual diet and other factors in both healthy people and those with diet-related diseases. To the best of our knowledge, this is the first questionnaire to evaluate dietary habits, lifestyle and nutrition knowledge whose test-retest reproducibility was assessed among people from all over the country, in a wide range of age, in healthy subjects and those suffering from chronic diseases. The objective of this study was to evaluate the reproducibility of a newly-developed questionnaire evaluating dietary habits, lifestyle and nutrition knowledge of adolescents and adults, including the assessment of indexes developed based on the questionnaire.

## 2. Methods

### 2.1. Participants and Study Design

A cross-sectional study was carried out by 5 scientific centres in Poland in 2014–2015. Two versions of the questionnaire were tested: interviewer-administered (IA-Q) and self-administered (SA-Q). The questionnaire was repeated after two weeks. The study protocol was registered and approved by the Bioethics Committee of the Faculty of Medical Sciences, University of Warmia and Mazury in Olsztyn on 17 June 2010, Resolution No. 20/2010). Written informed consent was provided by adult participants and parents or legal guardians of under-aged participants (<18 years).

Two groups of participants were recruited (non-randomly). They were labelled as healthy, i.e., subjects without diagnosed chronic diseases (self-declared by respondent), and outpatients, i.e., subjects with diagnosed chronic diseases (by a physician). Healthy subjects were selected non-randomly across Poland covering rural and urban areas and respondents of varied socioeconomic status. Outpatients came from the Central-West part of Poland (Wielkopolska region) and were recruited from four selected hospital clinics: metabolic diseases (overweight/obese outpatients with the Body Mass Index (BMI) ≥ 25.0 kg/m^2^), hypertension (hypertensive outpatients, >140/90 mmHg), diabetic (outpatients with diabetes mellitus type 1), gastrological (outpatients with inflammatory bowel disease, lack or slight tightening by the Crohn’s disease activity index (CDAI)). The recruitment of outpatients for the study was conducted in parallel with the project entitled ‘New bioactive food with designed functional properties’ carried out by the Poznan University of Life Sciences in Poland (No. POIG 01.01.02-00-061/09). 

**Figure 1:**
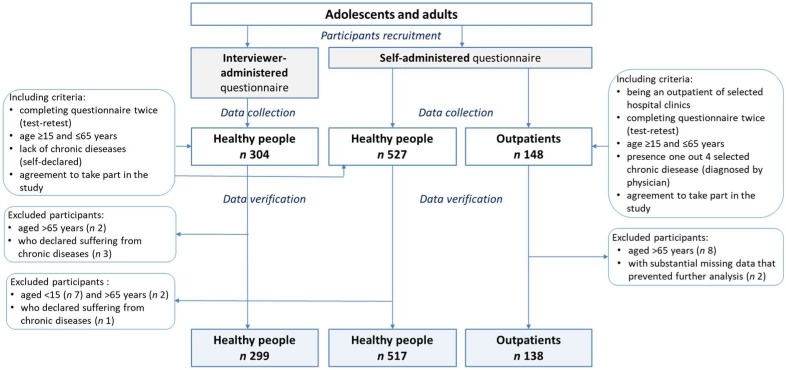
Study design and sample size.

Initially, 979 respondents participated in the study and completed the questionnaire twice (Figure 1). The database was verified and 25 subjects (2.5% of the initial sample) were excluded due to: age of respondents under 15 years (*n* 7) or over 65 years (*n* 12), a chronic disease declared by a respondent from healthy group (*n* 4), substantial missing data that prevented further analysis (*n* 2). Finally, in total 954 subjects aged 15-65 years (53.9% females) participated in the study. There were three subsamples: an interviewer-administered questionnaire (IA-Q) in healthy subjects (*n* 299), a self-administered questionnaire (SA-Q) in healthy subjects (*n* 517) and SA-Q in outpatients (*n* 138). The subsample of outpatients were respondents diagnosed with overweight/obesity (16%), inflammatory bowel disease (31%), hypertension (31%) and diabetes mellitus type 1 (22%).

### 2.2. The Dietary Habits and Nutrition Beliefs Questionnaire (KomPAN)

The questionnaire was developed in 2014 in two versions: interviewer-administered and self-administered [16]. The questionnaire contains 4 sections (Table S1):

- dietary habits (11 questions; 3 of them (no.: 10, 12, 17) were multiple response questions and each response category was encoded in the database and analysed as an independent dichotomous question with no/yes answers–the respondent’s choice of a given category was coded in the database as the answer ‘yes’, while no selection of the given category as the answer ‘no’),
- food frequency consumption (33 questions),
- nutrition beliefs (25 questions),
- lifestyle and personal data (14/30 questions were analysed).

Two diet quality scores (Pro-Healthy-Diet-Index-10 (pHDI-10), Non-Healthy-Diet-Index-14 (nHDI-14)), nutrition knowledge score and physical activity level were determined using the questionnaire [16]. 

Food frequency consumption was evaluated in 6 categories (from ‘never’ (1) to ‘few times a day’ (6)), assessing the habitual consumption of 33 food items over the past year. For each food item, the categories of frequency consumption were converted to values reflected daily frequency consumption (the range: 0–2 times/day) [16]. Diet quality scores were constructed a priori as a sum of the daily frequency consumption of foods: (i) pHDI-10 included 10 items representing pro-healthy food groups (no.: 23, 25, 31–33, 37, 38, 40, 42–43; the total score range: 0–20 points), (ii) nHDI-14 included 14 items representing unhealthy food groups (no.: 22, 24, 26–29, 34–36, 44, 46, 51–52, 54; the total score range: 0–28 points), and converted to unify the total score range to 0–100 points for each of the diet quality scores (Table S2). Both diet quality scores were then divided into three a priori categories: low (0–33 points), moderate (34–66 points), high (67–100 points), as well as those analysed in tertiles.

Nutrition knowledge was evaluated using 25 statements with 3 response categories (with assigned points): true (1 point), false or unsure (0 points) [16]. The total nutrition knowledge score was calculated as a sum of points (the range: 0–25 points). The nutrition knowledge level was categorised as follows: insufficient (0–8 points), sufficient (9–16 points), good knowledge (17–25 points). 

The questionnaire contains two questions concerning physical activity: physical activity at work/school and physical activity at leisure time [16]. Each of the questions had 3 response categories: low, moderate, high physical activity. The physical activity level was determined by combining these two questions categories and assessed in 3 categories: low, moderate and high physical activity (Table 1).

### 2.3. Statistical Analysis

The normality of variables was checked by the Kolmogorov–Smirnov test. Comparison analysis between the three subsamples for variables of the sample characteristics was performed using Kruskal–Wallis test (continuous variable) and χ2 test (categorical variables). 

To assess test-retest reproducibility of the questionnaire several analysis were used: (i) comparing the mean values (Wilcoxon signed-rank test), (ii) cross-classification analysis, (iii) calculation of the kappa statistic. Mean values and interquartile ranges were calculated for daily frequency consumption of all 33 food items, both diet quality scores and nutrition knowledge score. Cross-classification analysis and the kappa statistic were performed for all items regarding dietary habits, food frequency consumption, lifestyle and indexes determined based on the questionnaire (the full list of analysed items from the questionnaire was shown in Table S1). The kappa values above 0.40 were adopted as indicating an acceptable agreement [17]. Moreover, the internal reliability of diet quality scores and nutrition knowledge score was evaluated by calculation of the Cronbach’s alpha, and the values ranged 0.70–0.90 were adopted as recommended [18].

Sample size calculation was based on means and standard deviations of three scores: nutrition knowledge score and two diet quality scores. A database from a previous survey using the questionnaire and covering 217 adults was used. Data was collected during the pilot study for the first version of the questionnaire in 2013–2014 (data not published). Assuming a two-sided significance level of 0.05 and 80% power to detect a 20% difference in mean values of the scores between data from the test and retest, a minimum sample size was 103 to 343, including 10% drop out rate (in retest) and 10% of missing data. The results of the calculation were compared with similar validation studies [19] and a literature review of Cade et al. [1]. 

The statistical analysis was conducted using STATISTICA software (version 12.0 PL; StatSoft Inc., OK, USA; StatSoft, Kraków, Poland) and p-values below 0.05 were considered statistically significant.

## 3. Results

### 3.1. Characteristics of the Participants

Characteristics of the study sample are shown in Table 2. About half of the subjects in all three study subsamples, i.e., IA-Q in healthy subjects, SA-Q in healthy subjects and SA-Q in outpatients, were females (51.2%, 56.1% and 51.4%, respectively). Most of the respondents declared average economic situation of family (72.5%, 73.7% and 71.0%, respectively), were permanently employed (54.7%, 51.6% and 76.1%, respectively) and had higher education (44.7%, 52.9% and 44.9%, respectively). Significant differences between study subsamples were found in age (*p* < 0.0001), place of residence (*p* < 0.0001), occupation status (*p* < 0.0001) and education level (*p* < 0.05). Compared to the study subsamples of healthy people, a significantly higher proportion of outpatients were characterised by older age, living in a city, having a permanent employment and lower secondary education level.

### 3.2. Food Frequency Consumption

The mean daily frequency consumption of most food items assessed in test-retest of the questionnaire was not significantly different: 29/33 food items for IA-Q in healthy subjects, 23/33 for SA-Q in healthy subjects and 30/33 for SA-Q in outpatients (Table 3). The highest values were obtained for water (in all subsamples), while the lowest was obtained for tinned meat (IA-Q), lard (SA-Q in healthy subjects) and energy drinks (SA-Q in outpatients). For IA-Q conducted in healthy subjects, the test-retest agreement of classification into the same category of food frequency consumption was on average 79.3% and ranged from 72.2% (fruit juices) to 91.6% (energy drinks) (Table 4); the kappa statistic ranged from 0.62 (cold meats, smoked sausages, hot-dogs) to 0.84 (energy drinks) (Table 5). For SA-Q conducted in healthy subjects the cross-classification agreement was on average 74.2% and ranged from 63.8% (vegetable oils, margarines, mixes of butter and margarines) to 84.7% (lard); the kappa statistic ranged from 0.55 (vegetable oils, margarines, mixes of butter and margarines) to 0.78 (alcoholic beverages). For SA-Q in outpatients, the cross-classification agreement was on average 59.2% and ranged from 42.0% (both fruit juices and white rice, white pasta, fine-ground groats) to 92.0% (energy drinks); the kappa statistic ranged from 0.15 (white rice, white pasta, fine-ground groats) to 0.66 (energy drinks) and was ≥0.40 for 20/33 food items. The gross misclassification (3 or more categories) of food frequency consumption was the highest for SA-Q conducted in outpatients and averaged 4.4% (max. 9.4% of subjects for both white bread and wholemeal bread), and the SA-Q and IA-Q in healthy subjects averaged 2.7% and 1.3%, respectively (max. 8.9% and 7.0% for sweetened hot beverages) (Table 4).

### 3.3. Other Dietary Habits

The percentage of subjects classified into the same category in test-retest of the IA-Q conducted in healthy subjects averaged 91.0%, for SA-Q in healthy subjects it averaged 87.5% and in outpatients it was 80.7% (Table 4). In all study subsamples, the cross-classification agreement was the highest for ‘type of heat treatment of meat–I don’t eat meat’ (100.0% for IA-Q in healthy subjects, 99.4% for SA-Q in healthy subjects, 100.0% for SA-Q in outpatients) and the lowest for ‘frequency of snacking between meals’ (76.9%, 70.3%, 34.1% respectively) (Table 4). For IA-Q conducted in healthy subjects, the kappa statistic ranged from 0.71 (frequency of snacking between meals, snacking–sweetened dairy beverages and desserts, type of heat treatment of meat–fried) to 1.00 (type of heat treatment of meat–I don’t eat meat); the SA-Q in healthy subjects ranged from 0.58 (snacking–sweetened dairy beverages and desserts) to 0.88 (sweetening hot beverages); the SA-Q in outpatients ranged from 0.18 (frequency of snacking between meals) to 1.00 (type of heat treatment of meat–I don’t eat meat) (Table 5).

### 3.4. Lifestyle

For IA-Q conducted in healthy subjects, the cross-classification agreement averaged 90.4% and ranged from 80.6% (self-assessment of diet during weekdays compared to weekend) to 98.7% (smoking currently); for SA-Q in healthy subjects averaged 87.4% and ranged from 76.7% (time spend watching TV or using a computer) to 96.1% (smoking currently); the SA-Q in outpatients averaged 75.7% and ranged from 63.8% (time spend watching TV or using a computer) to 96.4% (smoking in the past) (Table 4). For IA-Q conducted in healthy subjects, the kappa statistic ranged from 0.68 (self-assessment of diet during weekdays compared to weekend) to 0.96 (smoking currently); for SA-Q in healthy subjects it was from 0.53 (type of alcohol usually consumed) to 0.87 (smoking currently) and for SA-Q in outpatients from 0.42 (type of alcohol usually consumed) to 0.93 (smoking in the past) (Table 5). 

### 3.5. Indexes Determined Based on the Questionnaire

Differences in mean values of indexes assessed in test and retest of the questionnaire were found for IA-Q conducted in healthy subjects (nHDI-14, nutrition knowledge), SA-Q in healthy subjects (nHDI-14) and SA-Q in outpatients (nutrition knowledge), while pHDI-10 was not significantly different in all study subsamples (Table 3). The cross-classification agreement was the highest for IA-Q in healthy subjects and the lowest for SA-Q in outpatients for all analysed indexes, except for nHDI-14 in which the highest cross-classification agreement was found for SA-Q in outpatients (Table 4). For IA-Q conducted in healthy subjects, the kappa statistic ranged from 0.65 (nHDI-14) to 0.83 (physical activity level); for SA-Q in healthy subjects it ranged from 0.58 (nHDI-14) to 0.76 (physical activity level) and the SA-Q in outpatients ranged from 0.43 (pHDI-10, nHDI-14 (in tertiles)) to 0.66 (nHDI-14) (Table 5). Regarding nutrition knowledge level, the kappa statistic ranged from 0.46 (for SA-Q in outpatients) to 0.73 (for IA-Q in healthy subjects).

The internal reliability of the pHDI-10 ranged from 0.66 (SA-Q in outpatients) to 0.71 (SA-Q in healthy subjects) for the nHDI-14 it ranged from 0.37 (SA-Q in outpatients) to 0.60 (IA-Q in healthy subjects) and for the nutrition knowledge score it ranged from 0.73 (SA-Q in outpatients) to 0.80 (both IA-Q and SA-Q in healthy subjects) (Table S2).

## 4. Discussion

Our findings indicated that the newly-developed questionnaire showed moderate to very good reproducibility for dietary, lifestyle and nutrition knowledge characteristics. The interviewer-administered questionnaire had higher reproducibility than the self-administered version of the questionnaire. Furthermore, the reproducibility of the self-administered questionnaire was higher in healthy people than in outpatients.

### 4.1. Interviewer-Administered vs. Self-Administered Questionnaire

To the best of our knowledge, the present study is the first study demonstrating the reproducibility of both interviewer- and self-administered versions of the questionnaire carried out in Poland. There were only a few similar studies from other countries reporting, for example, a comparison of a FFQ administered in various ways [20] or a validation of both interviewer-administered and self-administered FFQs against a reference method [21]. However, usually only one version of the questionnaire was the subject of research conducted in Poland [22,23,24,25] or other European countries [8,9,10,12]. According to a review of validation studies, 29% of FFQs were interviewer-administered and 71% of FFQs were self-administered [1]. In the present study, the interviewer-administered questionnaire demonstrated better reproducibility than the self-administered questionnaire, but it is important to take into account that engaging trained interviewers usually is more time-consuming and often involves higher research costs [1,26]. 

### 4.2. Food Frequency Consumption 

Cross-classification analysis showed a good-to-very good percentage of correct classification in test and retest of both interviewer- and self-administered versions of the questionnaire conducted in healthy subjects. In outpatients, the cross-classification analysis demonstrated a lower degree of reproducibility of the self-administered questionnaire. In this subsample, the percentage of subjects classified in the same category in test and retest for some variables was below 50% and gross misclassification was higher than was obtained among healthy people. Similarly, for both versions of the questionnaire tested in healthy people, the kappa values were above 0.40 for all analysed variables, as it is recommended and indicates an acceptable agreement [17]. Kappa values below 0.40 were found only in outpatients for about one-third of food items (13/33). Low reproducibility was noted for food items such as: ‘white rice, white pasta, fine-ground groats’, ‘vegetable oils, margarines, mixes of butter and margarines’, ‘fruit juices’ and ‘wholemeal bread’, while high reproducibility was observed for food items such as: ‘energy drinks’, ‘alcoholic beverages’, ‘lard’, ‘fish’.

The lower reproducibility of some food items may be due to difficulties in estimating the frequency of consumption. Foods consumed with low frequency seem to be easier to report than foods consumed with high frequency. It is easier to recall and accurately determine the consumption frequency of food items consumed never, or almost never, than estimating a more specific frequency for foods consumed more often. Similar to our findings, in other studies higher agreement was found for rarely-consumed foods (e.g., fish, avocado, reindeer meat) [9,12], while lower agreement was observed for frequently-consumed foods (e.g., white bread, whole-grain bread, potatoes) [9] or unspecific questions (e.g., ‘other vegetables’) [12]. Some difficulties in the consumption frequency estimation of regularly consumed foods have been noted in adolescents [9]. The consumption frequency may also be more difficult to assess by a respondent when several foods are listed in one question compared to single-listed foods [9]. Since this tool is a self-report method, the social desirability bias cannot be ruled out and could also affect the responses given by the respondents [4]. It was stated that obese, unhealthy or sporty people are particularly vulnerable to this bias and may avoid reporting on the consumption of non-recommended foods [27].

The lower reproducibility of the questionnaire in outpatients than in healthy people can be explained by within-subject variation, mental aspects and nutrition knowledge [3,4]. First, food consumption and other dietary habits may be characterised by higher day-to-day variation in people suffering from chronic diseases and trying to change their diet [3]. Balancing between right and wrong food choices may be reflected in greater variation of responses in the questionnaire. Outpatients may also be more prone to modify their dietary and lifestyle behaviours even within a relatively short, two-week interval between administrations of the questionnaire, and this could reduce the reproducibility. Secondly, outpatients were asked to fill out the questionnaire directly after visiting the doctor, so they could be more concerned about their health and/or tired of waiting for the visit, and consequently less focused on completing the questionnaire. Furthermore, outpatients had slightly lower nutrition knowledge than healthy people (12.9 vs. 13.3 points, respectively for the first administration of the self-administered questionnaire). Thus, taking into account all three possible causes, it can be assumed that the use of questionnaire, in both self- or interviewer-administrated versions, will be more burdened with uncertainty in people suffering from chronic diseases than in healthy people. 

### 4.3. Other Dietary Habits

The findings demonstrated that reporting behaviours that do not apply to a respondent, such as ‘type of heat treatment of meat–I don’t eat meat’ or ‘type of water consumed–I don’t drink water’, were characterised by higher reproducibility than questions requiring a more specific response. A high level of reproducibility was also observed for questions representing routine behaviours that are easy to recall, such as ‘sweetening hot beverages’, with relatively higher agreement in healthy subjects than in outpatients. Another example of such a question is ‘type of milk and dairy beverages (by fat content)’, with better classification agreement in healthy subjects than in outpatients (approx. 88% or 93% vs. 75%). Similarly, good reproducibility was found for questions such as ‘type of milk’ (83%) or ‘type of dairy products’ (79%) in a study conducted among Danish adolescents using self-administered questionnaire [9]. The lowest reproducibility was noted for ‘frequency of snacking between meals’, showing over two times higher classification agreement in healthy than in outpatients (approx. 70% or 77% vs. 34%). The agreement assessed by the kappa statistic was not acceptable only for some variables in outpatients (for 5/25 variables kappa was below 0.40 [17]). Possible reasons for the lower reproducibility of results regarding other dietary habits observed in outpatients compared with healthy subjects are similar to those given when discussing the reproducibility of the frequency of food consumption (Section 4.2). Therefore, our findings suggest the need for cautious interpretation of the variables for which relatively low reproducibility was demonstrated, especially in future studies among people with chronic diseases. 

### 4.4. Diet Quality Scores

Diet quality scores were developed based on the 24 items from the food frequency consumption section of the questionnaire and aimed at assessing healthy (pHDI-10) and unhealthy (nHDI-14) dietary behaviours separately [16]. The food items were chosen based on the literature review [28,29,30,31,32,33,34]. The same range of the total score (0–100) has been applied in other diet quality scores [7]. In general, the test-retest reproducibility of the pHDI-10 was better than demonstrated for the nHDI-14. Significant differences in the mean values of the nHDI-14 were found in repeat administration of the questionnaire in healthy subjects. Kappa values were acceptable but lower for nHDI-14 (0.43–0.66) than for pHDI-10 (0.43–0.80). The cross-classification analysis showed a high degree of reproducibility for both diet quality scores, but the agreement was better when the scores were analysed in the three a priori categories (83–99%) than in tertiles (62–83%).

The cross-classification agreement for a total score of other diet quality scores was lower than in the present study [6,7], with the same two-week interval between two administrations of the questionnaire in one of the studies [7] and longer period of time (5 weeks) used in the other research [6]. The degree of reproducibility measured by the kappa statistic among healthy individuals in the present study was also higher than found for the New Nordic Diet score tested in parents of toddlers [35] and a diet score including physical activity developed for Norwegian adolescents [5].

### 4.5. Nutrition Knowledge

Nutrition knowledge was assessed in two ways, i.e., using a score based on 25 questions (each with 3 response categories) and a single question (with 4 response categories) [16]. Our findings showed that for both measures, the degree of reproducibility was similar in healthy subjects, regardless of the mode of questionnaire administration (83–87%), but lower in outpatients (70–75%). It is noteworthy that a similar reproducibility was demonstrated for a 25-item nutrition knowledge test (the objective evaluation) and for a single question on nutrition knowledge (the self-assessment). Therefore, the reproducibility of nutrition knowledge measures seems to be influenced more by the respondents’ health status than by the way of assessment or the mode of questionnaire administration.

A high test-retest reproducibility of questionnaires to assess nutrition knowledge was demonstrated in other studies carried out in Italian adolescents [36], Turkish students [37], UK adults [38,39] or in Norwegian adults with obesity [40]. The degree of reproducibility of the nutrition knowledge questionnaire developed for obese adults was higher than demonstrated for nutrition knowledge measures in the present study in outpatients [40]. It was found that the nutrition knowledge score was significantly lower in adults reporting poor health status than in those reporting better health [38]. Since the time between test and retest can have some influence on the reproducibility of nutrition knowledge questionnaire [41], in the present study, a two-week interval was chosen similarly to other reproducibility studies regarding nutrition knowledge [37,38,39]. Such a period of time was expected to be long enough to forget previous responses and avoid recall bias, but short enough to avoid real changes in nutrition knowledge.

### 4.6. Lifestyle

Considering lifestyle behaviours, the highest reproducibility was found for dichotomous questions such as ‘smoking currently’ (healthy subjects) and ‘smoking in the past’ (outpatients), while the lowest classification agreement was demonstrated for ‘time spent watching TV or using a computer’ (a question with 6 response categories) using the self-administered questionnaire. Therefore, these findings confirm that dichotomous questions are easier to answer and are more reliable than multiple choice questions regarding lifestyle. Furthermore, the test-retest agreement of classification for physical activity level was very good in healthy subjects (approx. 87% or 91%) and slightly lower in outpatients (approx. 79%). Similar results were found for the types of physical activity–PA at work/school (70–91%) and PA at leisure time (73-87%). In other studies, very good classification agreement in test and retest of a self-administered FFQ was found for the question regarding doing sport exercises in spare time (98%) [9]. Despite some differences in the reproducibility observed in the present study, the agreement assessed by the kappa statistic for all variables regarding lifestyle was acceptable (above 0.40) [17], irrespective of the mode of questionnaire administration or the health status of respondents and, therefore, this tool can be recommended for use in epidemiological studies. 

### 4.7. Strengths and Limitations

Several strengths of this reproducibility study should be emphasised for future research. Firstly, the study was conducted in a large sample of men and women, adolescents and adults. Reproducibility and validation studies conducted previously among Poles were carried out in smaller samples [22,23,24,25,42], included solely young women or FFQs were developed for specific nutrient intake only [22,23,24,25]. Secondly, two diet quality scores have been developed based on the questionnaire [16] and the internal reliability and test-retest reproducibility of the diet quality scores were evaluated in the present study. As *a priori*-derived dietary patterns, the diet quality scores represent a complementary approach for evaluating the relationship between diet and health outcomes, capturing the synergistic effects of a combination of consumed foods [32,43,44]. Food-based scores may be especially useful in nutrition education interventions or clinical settings [32,43,45]. Moreover, the diet quality scores allow shortening the food frequency consumption section of the questionnaire to 24 of 33 items. Reducing the questionnaire length can be beneficial for future research when lower cost and less interviewer or respondent burden is required and may lead to higher response rate [46]. Next, in the present study, both interviewer- and self-administered versions of the questionnaire were tested, providing the key information to make an optimal choice in planning future studies. Furthermore, the study was carried out in healthy subjects and outpatients, allowing for the use of this questionnaire also in case-control studies. The reproducibility was assessed using several methods of statistical analysis, providing a comprehensive insight and strengthening the conclusions. According to a literature review of Lombard et al. [47], combinations of two or three statistical tests were most commonly applied in validation studies.

However, a few limitations should be considered when drawing the conclusions from the findings of the present study. Firstly, an interviewer-administered version of the questionnaire was tested solely in a group of healthy people. Therefore, for people suffering for some chronic diseases, only indirect inference about the reproducibility of the interviewer-administered questionnaire is possible, based on the results obtained for the self-administered questionnaire. Moreover, since portion-size questions were not included in food frequency consumption section of the questionnaire, data on the energy and nutritional value of the habitual diet are not possible to obtain. Nevertheless, the FFQs are mainly designed to rank individuals according to their usual consumption of foods or nutrient intake than to estimate the level of intake [1,2] and our findings showed that, for example, the test-retest reproducibility of ranking individuals to tertiles of diet quality scores evaluated based on the questionnaire was good.

In conclusion, the present study showed moderate-to-very good reproducibility of the Dietary Habits and Nutrition Beliefs Questionnaire (KomPAN) which is the first such complex tool developed in Poland to evaluate and monitor changes in dietary habits, lifestyle and nutrition knowledge from adolescence to old age, in groups of people with different health status. The results showed that the reproducibility of the interviewer-administered questionnaire was better than its self-administered version and, for the self-administered questionnaire, the reproducibility was better in healthy people than in outpatients. The development of a web-based English language version of the questionnaire may facilitate its application in large-scale studies across culturally similar countries of Europe. However, comparing the questionnaire with another dietary assessment method or biomarkers is required in future research. 

## Figures and Tables

**Table 1:** Combining two questions concerning physical activity [16].

Physical Activity at Work/School	Physical Activity at Leisure Time		
	Low	Moderate	High
Low	Low	Low	Moderate
Moderate	Low	Moderate	Moderate
High	Moderate	Moderate	High

**Table 2:** Characteristics of the participants.

	Interviewer-Administered Questionnaire		Self-Administered Questionnaire				*p*
	Healthy People		Healthy People		Outpatients #		
	*n*	%	*n*	%	*n*	%	
Sample size	299	100.0	517	100.0	138	100.0	
Sex							
male	146	48.8	227	43.9	67	48.6	ns
female	153	51.2	290	56.1	71	51.4	
Age (years) †		36.3 (14.6)		30.2 (15.0)		41.9 (12.0)	****
Age (years)							
15–24	100	33.4	291	56.3	18	13.1	****
25–44	103	34.5	107	20.7	62	44.9	
45–65	96	32.1	119	23.0	58	42.0	
Place of residence ‡							
village	74	24.8	185	35.8	26	19.0	****
small town (<20,000 inhabitants)	65	21.8	81	15.7	17	12.4	
town (20,000–100,000 inhabitants)	58	19.5	112	21.6	24	17.5	
city (>100,000 inhabitants)	101	33.9	139	26.9	70	51.1	
Economic situation of family ‡							
below average	15	5.0	30	5.8	12	8.7	ns
average	216	72.5	381	73.7	98	71.0	
above average	67	22.5	106	20.5	28	20.3	
Economic situation of household							
we live modestly or very modestly	19	6.4	27	5.2	8	5.8	ns
we live normally	123	41.1	210	40.6	75	54.4	
we live relatively wealthy	137	45.8	241	46.6	46	33.3	
we live very wealthy	20	6.7	39	7.6	9	6.5	
Occupation status (adults) ‡							
retired/receiving a disability living allowance	17	5.9	22	6.3	8	5.8	****
unemployed/maternity leave/other	63	22.0	121	34.7	14	10.1	
temporary employed	50	17.4	26	7.4	11	8.0	
permanently employed	157	54.7	180	51.6	105	76.1	
Education level (adults) ‡							
primary	32	11.2	23	6.3	5	3.6	*
lower secondary	26	9.1	23	6.3	17	12.3	
upper secondary	100	35.0	126	34.5	54	39.2	
higher	128	44.7	193	52.9	62	44.9	

# overweight/obesity (16% of the outpatients sample), inflammatory bowel disease (31%), hypertension (31%), diabetes mellitus type 1 (22%). †: mean value (standard deviation). ‡: the sample size vary due to the lack of data for those variables. *p*: significance level of Kruskal–Wallis test (continuous variable) and χ^2^ test (categorical variables): * *p* <0.05, **** *p* < 0.0001, ns: not statistically significant.

**Table 3:** Mean values and interquartile ranges (Q25–Q75) in test and retest of the Dietary Habits and Nutrition Beliefs Questionnaire (KomPAN).

No.	Questionnaire Items	Interviewer-Administered Questionnaire Healthy People (*n* 299)					Self-Administered Questionnaire Healthy People (*n* 517)					Self-Administered Questionnaire Outpatients # (*n* 138)				
		Test		Retest		*p*	Test		Retest		*p*	Test		Retest		*p*
		Mean	Q25–Q75	Mean	Q25–Q75		Mean	Q25–Q75	Mean	Q25–Q75		Mean	Q25–Q75	Mean	Q25–Q75	
	Indexes (in points)															
	pHDI-10 †	24.7	(14.9–33.8)	24.4	(15.1–32.4)	ns	24.9	(15.6–31.3)	24.3	(15.2–30.9)	ns	22.3	(14.4–29.5)	21.8	(13.8–28.9)	ns
	nHDI-14 ‡	18.0	(12.1–23.4)	17.4	(11.4–22.1)	*	18.5	(11.8–23.9)	17.6	(10.9–22.9)	***	16.5	(12.3–20.9)	15.9	(11.1–20.6)	ns
	Nutrition knowledge score §	14.1	(11.0–18.0)	14.4	(11.0–18.0)	*	13.3	(10.0–17.0)	13.5	(10.0–17.0)	ns	12.9	(10.0–15.0)	13.4	(11.0–16.0)	**
	Food frequency consumption ¶ (times/day)															
53	Water	1.17	(0.50–2.00)	1.17	(0.50–2.00)	ns	1.27	(0.50–2.00)	1.23	(0.50–2.00)	ns	1.38	(0.50–2.00)	1.29	(0.50–2.00)	ns
43	Vegetables	1.03	(0.50–2.00)	1.00	(0.50–2.00)	ns	0.92	(0.50–1.00)	0.92	(0.50–1.00)	ns	0.82	(0.50–1.00)	0.73	(0.50–1.00)	*
42	Fruit	0.99	(0.50–2.00)	0.97	(0.50–2.00)	ns	0.96	(0.50–2.00)	0.94	(0.50–1.00)	ns	0.72	(0.50–1.00)	0.72	(0.50–1.00)	ns
50	Sweetened hot beverages	0.92	(0.06–2.00)	0.96	(0.06–2.00)	ns	1.02	(0.06–2.00)	0.97	(0.06–2.00)	ns	0.90	(0.00–2.00)	0.95	(0.00–2.00)	ns
22	White bread	0.85	(0.14–2.00)	0.79	(0.14–1.00)	*	0.91	(0.14–2.00)	0.84	(0.14–1.00)	**	0.94	(0.50–2.00)	0.89	(0.14–2.00)	ns
35	Cold meats, smoked sausages, hot-dogs	0.66	(0.50–1.00)	0.60	(0.14–0.50)	*	0.68	(0.14–1.00)	0.62	(0.14–1.00)	**	0.62	(0.50–1.00)	0.62	(0.14–1.00)	ns
31	Milk	0.65	(0.14–1.00)	0.63	(0.14–1.00)	ns	0.68	(0.14–1.00)	0.62	(0.14–1.00)	**	0.58	(0.06–1.00)	0.55	(0.06–1.00)	ns
28	Butter	0.64	(0.06–1.00)	0.64	(0.06–1.00)	ns	0.72	(0.06–1.00)	0.65	(0.06–1.00)	***	0.97	(0.50–2.00)	0.89	(0.14–2.00)	ns
30	Vegetable oils, margarines, mixes of butter and margarines	0.60	(0.14–1.00)	0.57	(0.06–1.00)	ns	0.48	(0.06–0.50)	0.49	(0.06–0.50)	ns	0.57	(0.14–1.00)	0.49	(0.06–0.50)	ns
23	Wholemeal bread	0.58	(0.06–1.00)	0.57	(0.06–1.00)	ns	0.57	(0.06–1.00)	0.54	(0.06–0.50)	*	0.57	(0.06–1.00)	0.52	(0.06–1.00)	ns
44	Sweets	0.57	(0.14–1.00)	0.54	(0.14–1.00)	ns	0.69	(0.14–1.00)	0.65	(0.14–1.00)	*	0.46	(0.06–0.50)	0.43	(0.06–0.50)	ns
34	Cheese	0.44	(0.14–0.50)	0.40	(0.14–0.50)	**	0.46	(0.14–0.50)	0.45	(0.14–0.50)	ns	0.39	(0.14–0.50)	0.40	(0.14–0.50)	ns
32	Fermented milk beverages	0.43	(0.14–0.50)	0.42	(0.14–0.50)	ns	0.50	(0.14–0.50)	0.47	(0.14–0.50)	ns	0.44	(0.06–0.50)	0.45	(0.14–0.50)	ns
37	White meat	0.41	(0.14–0.50)	0.42	(0.14–0.50)	ns	0.43	(0.14–0.50)	0.43	(0.14–0.50)	ns	0.44	(0.50–0.50)	0.50	(0.50–0.50)	*
41	Potatoes (excluding chips and crisps)	0.40	(0.14–0.50)	0.42	(0.14–0.50)	ns	0.44	(0.14–0.50)	0.44	(0.14–0.50)	ns	0.35	(0.14–0.50)	0.35	(0.14–0.50)	ns
48	Fruit juices	0.36	(0.06-0.50)	0.37	(0.06–0.50)	ns	0.48	(0.06–0.50)	0.46	(0.06–0.50)	ns	0.61	(0.14–1.00)	0.54	(0.06–0.50)	*
24	White rice, white pasta, fine-ground groats	0.35	(0.14–0.50)	0.35	(0.06–0.50)	ns	0.28	(0.06–0.50)	0.28	(0.06–0.50)	ns	0.29	(0.06–0.50)	0.31	(0.14–0.50)	ns
27	Fried foods	0.35	(0.14–0.50)	0.36	(0.14–0.50)	ns	0.35	(0.14–0.50)	0.32	(0.14–0.50)	**	0.28	(0.06–0.50)	0.26	(0.06–0.50)	ns
39	Eggs	0.33	(0.14–0.50)	0.32	(0.14–0.50)	ns	0.29	(0.14–0.50)	0.30	(0.14–0.50)	ns	0.28	(0.14–0.50)	0.28	(0.14–0.50)	ns
36	Red meat	0.32	(0.06–0.50)	0.32	(0.06–0.50)	ns	0.26	(0.06–0.50)	0.26	(0.06–0.50)	ns	0.26	(0.06–0.50)	0.27	(0.06–0.50)	ns
33	Fresh cheese curd products	0.30	(0.06–0.50)	0.30	(0.06–0.50)	ns	0.39	(0.06–0.50)	0.36	(0.06–0.50)	ns	0.32	(0.06–0.50)	0.33	(0.14–0.50)	ns
51	Sweetened beverages	0.30	(0.06–0.50)	0.29	(0.00–0.50)	ns	0.32	(0.06–0.50)	0.30	(0.06–0.50)	ns	0.15	(0.00–0.14)	0.13	(0.00–0.06)	ns
25	Buckwheat, oats, wholegrain pasta, other coarse-ground groats	0.29	(0.06–0.50)	0.28	(0.06–0.50)	ns	0.25	(0.06–0.50)	0.25	(0.06–0.50)	ns	0.29	(0.06–0.50)	0.30	(0.06–0.50)	ns
47	Tinned vegetables	0.17	(0.06–0.14)	0.17	(0.06–0.14)	ns	0.16	(0.00–0.14)	0.17	(0.06–0.14)	ns	0.22	(0.06–0.50)	0.23	(0.06–0.50)	ns
54	Alcoholic beverages	0.17	(0.06–0.14)	0.15	(0.06–0.14)	ns	0.16	(0.00–0.14)	0.18	(0.00–0.14)	ns	0.10	(0.06–0.14)	0.09	(0.00–0.06)	ns
38	Fish	0.16	(0.06–0.14)	0.16	(0.06–0.14)	ns	0.16	(0.06–0.14)	0.17	(0.06–0.14)	ns	0.17	(0.06–0.14)	0.17	(0.06–0.14)	ns
49	Vegetable juices, fruit and vegetable juices	0.16	(0.00–0.14)	0.19	(0.00–0.14)	ns	0.16	(0.00–0.14)	0.20	(0.00–0.14)	**	0.15	(0.00–0.14)	0.15	(0.00–0.14)	ns
26	Fast foods	0.13	(0.06–0.14)	0.12	(0.06–0.14)	ns	0.10	(0.06–0.06)	0.10	(0.06–0.06)	ns	0.05	(0.00–0.06)	0.05	(0.00–0.06)	ns
29	Lard	0.11	(0.00–0.06)	0.12	(0.00–0.06)	ns	0.05	(0.00–0.06)	0.08	(0.00–0.06)	*	0.04	(0.00–0.06)	0.03	(0.00–0.06)	ns
40	Pulse-based foods	0.11	(0.06–0.14)	0.12	(0.06–0.14)	ns	0.13	(0.06–0.14)	0.15	(0.06–0.14)	*	0.09	(0.00–0.06)	0.08	(0.06–0.06)	ns
52	Energy drinks	0.10	(0.00–0.06)	0.10	(0.00–0.06)	ns	0.12	(0.00–0.06)	0.12	(0.00–0.06)	ns	0.02	(0.00–0.00)	0.01	(0.00–0.00)	ns
45	Instant soups, ready-made soups	0.08	(0.00–0.06)	0.09	(0.00–0.06)	ns	0.07	(0.00–0.06)	0.09	(0.00–0.06)	ns	0.20	(0.00–0.14)	0.23	(0.00–0.50)	ns
46	Tinned meat	0.07	(0.00–0.06)	0.07	(0.00–0.06)	*	0.06	(0.00–0.06)	0.08	(0.00–0.06)	ns	0.04	(0.00–0.06)	0.07	(0.00–0.06)	ns

No.: item number in the questionnaire. #: overweight/obesity (16% of the outpatients sample), inflammatory bowel disease (31%), hypertension (31%), diabetes mellitus type 1 (22%). *p*: significance level of Wilcoxon signed-rank test: * *p* < 0.05, ** *p* < 0.01, *** *p* < 0.001, ns: not statistically significant. † pHDI-10: Pro-Healthy-Diet-Index-10 including 10 questions no.: 23, 25, 31–33, 37, 38, 40, 42–43 (the total score range: 0–100). ‡ nHDI-14: Non-Healthy-Diet-Index-14 including 14 questions no.: 22, 24, 26–29, 34–36, 44, 46, 51–52, 54 (the total score range: 0–100). § Nutrition knowledge score: evaluated based on 25 questions no. 55–79, with 3 response categories: true, false, unsure (the total score range: 0–25). ¶ expressed as daily frequency consumption (range: 0–2).

**Table 4:** Agreement and misclassification in test and retest of the Dietary Habits and Nutrition Beliefs Questionnaire (KomPAN) (%).

No.	Questionnaire Items	Cat.	Interviewer-Administered Questionnaire Healthy People (*n* 299)				Self-Administered Questionnaire Healthy People (*n* 517)				Self-Administered Questionnaire Outpatients # (*n* 138)			
			Total Agreement	Misclassification			Total Agreement	Misclassification			Total Agreement	Misclassification		
				±1 Cat.	±2 Cat.	±3 Cat. or More		±1 Cat.	±2 Cat.	±3 Cat. or More		±1 Cat.	±2 Cat.	±3 Cat. or More
	Indexes													
	nHDI-14 (a priori categories) †	3	97.3	2.7	0.0		96.1	3.9	0.0		98.6	1.4	0.0	
	pHDI-10 (a priori categories) ‡	3	93.0	7.0	0.0		90.1	9.9	0.0		83.3	16.7	0.0	
	Physical activity level §	3	91.3	8.7	0.0		87.4	12.4	0.2		79.4	20.6	0.0	
	Nutrition knowledge level ¶	3	83.9	16.1	0.0		83.0	16.2	0.8		75.4	24.6	0.0	
	pHDI-10 (tertiles) ¥	3	83.3	15.7	1.0		77.6	20.3	2.1		63.0	34.1	2.9	
	nHDI-14 (tertiles) ¥	3	77.6	20.7	1.7		77.4	19.7	2.9		62.3	34.1	3.6	
	Dietary habits (mean value of 25 items)		91.0	8.5	1.1	0.7	87.5	11.1	2.9	2.5	80.7	16.9	4.4	4.5
12.6	Type of heat treatment of meat–I don’t eat meat	2	100.0	0.0			99.4	0.6			100.0	0.0		
17.1	Type of water consumed–I don’t drink water	2	97.7	2.3			97.9	2.1			95.7	4.3		
12.3	Type of heat treatment of meat–grilled	2	95.3	4.7			89.7	10.3			91.3	8.7		
17.4	Type of water consumed–flavoured water	2	95.0	5.0			90.3	9.7			92.0	8.0		
17.2	Type of water consumed–still water	2	94.3	5.7			93.0	7.0			87.0	13.0		
15	Sweetening hot beverages	4	94.0	5.7	0.3	0.0	91.9	6.4	1.5	0.2	83.3	14.5	1.4	0.7
10.7	Snacking–nuts, almonds, seeds	2	93.6	6.4			88.4	11.6			89.9	10.1		
10.6	Snacking–savoury snacks	2	93.3	6.7			87.8	12.2			86.2	13.8		
17.3	Type of water consumed–sparkling water	2	93.3	6.7			91.3	8.7			89.1	10.9		
11	Type of milk and dairy beverages (by fat content)	3	93.2	6.5	0.3		88.0	10.8	1.2		74.6	23.0	2.4	
13	Type of bread spread	7	93.0	4.3	1.3	1.3	85.4	5.1	3.3	6.3	86.2	5.1	1.4	7.2
10.2	Snacking–vegetables	2	92.0	8.0			87.2	12.8			83.3	16.7		
14	Type of frying fat	6	91.6	5.4	2.3	0.7	86.4	7.4	3.3	2.9	85.5	8.7	4.3	1.4
10.1	Snacking–fruit	2	91.3	8.7			89.9	10.1			65.9	34.1		
10.3	Snacking–unsweetened dairy beverages and desserts	2	90.6	9.4			83.9	16.1			74.6	25.4		
10.4	Snacking–sweetened dairy beverages and desserts	2	90.3	9.7			82.6	17.4			87.7	12.3		
12.2	Type of heat treatment of meat–stewed	2	89.0	11.0			89.5	10.5			76.1	23.9		
10.5	Snacking–sweet snacks	2	88.6	11.4			84.5	15.5			79.0	21.0		
16	Adding salt to meals	3	88.6	11.1	0.3		86.8	11.8	1.4		82.6	15.9	1.4	
12.1	Type of heat treatment of meat–boiled	2	87.6	12.4			85.3	14.7			75.4	24.6		
12.5	Type of heat treatment of meat–fried	2	87.3	12.7			86.6	13.4			77.5	22.5		
12.4	Type of heat treatment of meat–roasted	2	86.3	13.7			85.9	14.1			75.4	24.6		
7	Number of meals a day	5	86.0	13.4	0.7	0.0	82.2	15.7	1.9	0.2	74.6	22.5	2.9	0.0
8	Regularity of consuming meals	3	86.0	13.7	0.3		83.8	15.3	1.0		71.7	24.6	3.6	
9	Frequency of snacking between meals	6	76.9	18.1	3.3	1.7	70.3	17.2	9.5	2.9	34.1	34.8	18.1	13.0
	Food frequency consumption (mean value of 33 items)		79.3	15.6	3.8	1.3	74.2	17.5	5.6	2.7	59.2	29.0	7.3	4.4
52	Energy drinks	6	91.6	7.0	1.0	0.3	84.5	9.9	3.3	2.3	92.0	8.0	0.0	0.0
29	Lard	6	89.0	7.7	1.3	2.0	84.7	9.7	3.3	2.3	84.1	11.6	0.7	3.6
46	Tinned meat	6	88.6	9.7	1.0	0.7	82.2	13.3	3.1	1.4	76.1	20.3	1.4	2.2
54	Alcoholic beverages	6	88.0	10.4	1.0	0.7	83.6	12.8	2.7	1.0	74.6	22.5	2.2	0.7
45	Instant soups, ready-made soups	6	84.9	14.0	0.7	0.3	81.8	13.7	3.1	1.4	51.4	20.3	5.8	22.5
38	Fish	6	83.9	14.4	1.0	0.7	78.5	16.8	3.7	1.0	65.2	31.9	2.9	0.0
26	Fast foods	6	83.6	13.7	2.3	0.3	82.0	14.0	3.3	0.8	73.2	26.1	0.7	0.0
41	Potatoes (excluding chips and crisps)	6	83.3	14.0	2.3	0.3	79.9	15.9	3.3	1.0	61.6	28.3	6.5	3.6
42	Fruit	6	81.6	13.7	3.7	1.0	72.3	19.5	5.6	2.5	57.2	35.5	5.1	2.2
53	Water	6	81.6	13.0	3.7	1.7	74.9	12.0	7.9	5.2	64.5	22.5	8.7	4.3
43	Vegetables	6	80.9	14.4	4.3	0.3	70.8	20.9	6.0	2.3	60.1	30.4	8.7	0.7
37	White meat	6	80.6	16.7	2.3	0.3	77.6	17.2	3.3	1.9	65.2	27.5	7.2	0.0
51	Sweetened beverages	6	80.6	14.0	4.0	1.3	77.0	15.3	5.4	2.3	59.4	26.8	10.1	3.6
50	Sweetened hot beverages	6	79.3	11.0	2.7	7.0	75.0	11.4	4.6	8.9	63.0	10.1	1.4	25.4
39	Eggs	6	78.9	17.4	3.0	0.7	74.9	21.3	2.9	1.0	64.5	34.1	1.4	0.0
40	Pulses-based foods	6	78.9	17.4	3.0	0.7	77.6	16.4	4.1	1.9	66.7	28.3	4.3	0.7
49	Vegetable juices, fruit and vegetable juices	6	78.6	14.4	5.0	2.0	70.2	19.5	5.4	4.8	54.3	33.3	4.3	8.0
36	Red meat	6	78.5	15.1	5.7	0.7	73.9	17.2	6.8	2.1	60.1	29.7	10.1	0.0
25	Buckwheat, oats, wholegrain pasta, other coarse-ground groats	6	78.2	16.8	3.0	2.0	69.2	22.2	6.0	2.5	48.6	31.9	15.9	3.6
24	White rice, white pasta, fine-ground groats	6	77.3	18.1	4.3	0.3	70.6	22.2	6.4	0.8	42.0	44.9	10.9	2.2
28	Butter	6	77.3	14.0	5.7	3.0	66.5	17.4	9.5	6.6	53.6	32.6	8.0	5.8
23	Wholemeal bread	6	76.9	15.7	5.0	2.3	72.3	19.3	5.8	2.5	43.5	34.1	13.0	9.4
32	Fermented milk beverages	6	76.3	20.1	3.0	0.7	69.4	21.5	7.2	1.9	58.7	32.6	7.2	1.4
34	Cheese	6	75.9	19.4	4.3	0.3	73.3	18.2	7.2	1.4	59.4	34.8	4.3	1.4
27	Fried foods	6	75.3	19.7	4.0	1.0	74.5	18.2	6.8	0.6	57.2	35.5	7.2	0.0
33	Fresh cheese curd products	6	74.9	21.7	3.3	0.0	70.0	21.7	6.0	2.3	50.7	38.4	7.2	3.6
44	Sweets	6	74.6	17.7	6.4	1.3	70.2	20.7	6.8	2.3	53.6	29.7	10.9	5.8
47	Tinned vegetables	6	74.2	20.4	4.3	1.0	67.7	22.4	7.4	2.5	52.9	36.2	9.4	1.4
30	Vegetable oils, margarines, mixes of butter and margarines	6	73.2	14.7	7.7	4.3	63.8	17.4	10.6	8.1	43.1	31.4	13.9	11.7
31	Milk	6	72.9	18.7	6.0	2.3	68.9	20.5	7.0	3.7	56.2	32.8	8.0	2.9
35	Cold meats, smoked sausages, hot-dogs	6	72.6	19.1	8.0	0.3	71.8	20.3	5.2	2.7	47.1	39.1	12.3	1.4
22	White bread	6	72.5	19.1	6.4	2.0	69.1	16.8	8.7	5.4	51.4	26.8	12.3	9.4
48	Fruit juices	6	72.2	21.4	5.7	0.7	69.8	20.7	6.6	2.9	42.0	30.4	18.8	8.7
	Lifestyle (mean value of 14 items)		90.4	8.9	0.6	0.6	87.4	10.5	1.8	1.5	75.7	21.1	3.0	1.5
85	Smoking currently	2	98.7	1.3			96.1	3.9			94.2	5.8		
80	Following a diet (currently)	3	98.0	0.7	1.3		95.2	1.4	3.5		65.9	21.0	13.0	
86	Smoking in the past	2	96.0	4.0			93.2	6.8			96.4	3.6		
84	Type of alcohol usually consumed	4	94.0	4.4	0.4	1.2	91.3	3.8	2.2	2.7	79.8	11.1	6.1	3.0
92	Self-assessment of health status compared to other people of the same age	3	92.6	7.0	0.3		90.5	9.1	0.4		76.1	22.5	1.4	
87	Sleep time on weekdays	3	92.0	8.0	0.0		90.1	9.7	0.2		79.0	21.0	0.0	
90	Physical activity at work/school	3	90.6	9.1	0.3		85.7	12.8	1.5		70.2	29.8	0.0	
94	Self-assessment of diet	4	90.6	9.4	0.0	0.0	89.7	8.7	1.4	0.2	78.3	21.0	0.7	0.0
88	Sleep time on weekends	3	90.0	9.0	1.0		85.0	14.0	1.0		77.5	21.0	1.4	
83	Eating out	6	87.0	11.4	0.7	1.0	81.6	14.0	2.9	1.6	66.7	28.3	3.6	1.4
93	Self-assessment of nutrition knowledge	4	87.0	12.7	0.3	0.0	82.8	15.5	1.5	0.2	70.3	27.5	2.2	0.0
91	Physical activity at leisure time	3	86.6	13.0	0.3		86.2	13.2	0.6		73.2	26.1	0.7	
89	Time spent watching TV or using a computer	6	81.9	15.7	1.3	1.0	76.7	15.7	4.7	2.9	63.8	27.5	5.8	2.9
95	Self-assessment of diet during weekdays compared to weekend	3	80.6	18.4	1.0		79.9	18.8	1.4		68.8	29.7	1.4	

No.: item number in the questionnaire. Cat.: number of response categories in the question. #: overweight/obesity (16% of the outpatients sample), inflammatory bowel disease (31%), hypertension (31%), diabetes mellitus type 1 (22%). † nHDI-14: Non-Healthy-Diet-Index-14, including 14 questions no.: 22, 24, 26–29, 34–36, 44, 46, 51–52, 54, evaluated in 3 categories: low (0–33 points), moderate (34–66 points), high (67–100 points). ‡ pHDI-10: Pro-Healthy-Diet-Index-10, including 10 questions no.: 23, 25, 31–33, 37, 38, 40, 42–43, evaluated in 3 categories: low (0–33 points), moderate (34–66 points), high (67–100 points). § Physical activity level: determined by combining two question, i.e., physical activity at work/school (3 categories) and physical activity at leisure time (3 categories), and assessed in 3 categories: low, moderate and high (i.e., high PA at work/school and high PA at leisure time). ¶ Nutrition knowledge level: evaluated based on 25 questions no. 55–79 (with 3 response categories: true, false, unsure) and assessed in 3 categories: insufficient (0–8 points), sufficient (9–16 points), good (17–25 points). ¥ tertiles of the diet quality indexes were determined separately for each study group in test and retest of the questionnaire.

**Table 5:** Kappa statistics for test and retest of the Dietary Habits and Nutrition Beliefs Questionnaire (KomPAN).

No.	Questionnaire Items	Cat.	Interviewer-Administered Questionnaire	Self-Administered Questionnaire	
			Healthy People	Healthy People	Outpatients #
	Sample size		299	517	138
	Indexes				
	Physical activity level †	3	0.83	0.76	0.52
	pHDI-10 (a priori categories) ‡	3	0.80	0.69	0.43
	pHDI-10 (tertiles) §	3	0.75	0.66	0.45
	Nutrition knowledge level ¶	3	0.73	0.71	0.46
	nHDI-14 (tertiles) §	3	0.66	0.66	0.43
	nHDI-14 (a priori categories) ¥	3	0.65	0.58	0.66
	Dietary habits				
12.6	Type of heat treatment of meat–I don’t eat meat	2	1.00	0.79	1.00
15	Sweetening hot beverages	4	0.91	0.88	0.74
13	Type of bread spread	7	0.90	0.78	0.76
17.2	Type of water consumed–still water	2	0.88	0.84	0.64
12.3	Type of heat treatment of meat–grilled	2	0.86	0.76	0.71
17.3	Type of water consumed–sparkling water	2	0.85	0.80	0.65
11	Type of milk and dairy beverages (by fat content)	3	0.84	0.75	0.38
10.1	Snacking–fruit	2	0.82	0.75	0.31
17.4	Type of water consumed–flavoured water	2	0.82	0.71	0.43
14	Type of frying fat	6	0.81	0.69	0.60
7	Number of meals a day	5	0.79	0.74	0.61
16	Adding salt to meals	3	0.79	0.77	0.65
17.1	Type of water consumed–I don’t drink water	2	0.78	0.72	0.64
10.5	Snacking–sweet snacks	2	0.77	0.68	0.58
10.6	Snacking–savoury snacks	2	0.77	0.67	0.43
10.7	Snacking–nuts, almonds, seeds	2	0.77	0.67	0.66
12.2	Type of heat treatment of meat–stewed	2	0.77	0.77	0.52
8	Regularity of consuming meals	3	0.76	0.73	0.55
10.3	Snacking–unsweetened dairy beverages and desserts	2	0.74	0.62	0.30
12.1	Type of heat treatment of meat–boiled	2	0.74	0.70	0.50
10.2	Snacking–vegetables	2	0.73	0.66	0.31
12.4	Type of heat treatment of meat–roasted	2	0.72	0.69	0.50
9	Frequency of snacking between meals	6	0.71	0.62	0.18
10.4	Snacking–sweetened dairy beverages and desserts	2	0.71	0.58	0.49
12.5	Type of heat treatment of meat–fried	2	0.71	0.69	0.55
	Food frequency consumption				
52	Energy drinks	6	0.84	0.74	0.66
54	Alcoholic beverages	6	0.83	0.78	0.61
46	Tinned meat	6	0.81	0.70	0.49
29	Lard	6	0.80	0.68	0.62
41	Potatoes (excluding chips and crisps)	6	0.76	0.69	0.40
42	Fruit	6	0.76	0.62	0.42
26	Fast foods	6	0.75	0.69	0.53
38	Fish	6	0.75	0.67	0.44
51	Sweetened beverages	6	0.75	0.71	0.40
43	Vegetables	6	0.74	0.59	0.40
45	Instant soups, ready-made soups	6	0.74	0.70	0.29
53	Water	6	0.74	0.63	0.45
50	Sweetened hot beverages	6	0.73	0.67	0.46
28	Butter	6	0.72	0.60	0.42
23	Wholemeal bread	6	0.71	0.66	0.31
25	Buckwheat, oats, wholegrain pasta, other coarse-ground groats	6	0.71	0.59	0.34
36	Red meat	6	0.71	0.65	0.44
49	Vegetable juices, fruit and vegetable juices	6	0.71	0.60	0.38
39	Eggs	6	0.70	0.63	0.44
37	White meat	6	0.69	0.63	0.27
24	White rice, white pasta, fine-ground groats	6	0.68	0.58	0.15
32	Fermented milk beverages	6	0.68	0.60	0.46
34	Cheese	6	0.67	0.62	0.40
40	Pulses-based foods	6	0.67	0.62	0.48
30	Vegetable oils, margarines, mixes of butter and margarines	6	0.66	0.55	0.26
31	Milk	6	0.66	0.61	0.44
33	Fresh cheese curd products	6	0.66	0.60	0.32
22	White bread	6	0.65	0.61	0.39
44	Sweets	6	0.65	0.62	0.39
27	Fried foods	6	0.64	0.62	0.40
47	Tinned vegetables	6	0.64	0.56	0.37
48	Fruit juices	6	0.64	0.61	0.28
35	Cold meats, smoked sausages, hot-dogs	6	0.62	0.61	0.19
	Lifestyle				
85	Smoking currently	2	0.96	0.87	0.73
86	Smoking in the past	2	0.92	0.85	0.93
80	Following a diet (currently)	3	0.91	0.76	0.48
87	Sleep time on weekdays	3	0.85	0.79	0.51
92	Self-assessment of health status compared to other people of the same age	3	0.85	0.79	0.61
90	Physical activity at work/school	3	0.84	0.76	0.46
88	Sleep time on weekends	3	0.83	0.73	0.57
83	Eating out	6	0.81	0.71	0.52
93	Self-assessment of nutrition knowledge	4	0.79	0.72	0.52
94	Self-assessment of diet	4	0.79	0.77	0.43
91	Physical activity at leisure time	3	0.78	0.77	0.51
89	Time spent watching TV or using a computer	6	0.77	0.69	0.54
84	Type of alcohol usually consumed	4	0.70	0.53	0.42
95	Self-assessment of diet during weekdays compared to weekend	3	0.68	0.66	0.49

No.: item number in the questionnaire. Cat.: number of response categories in the question. #: overweight/obesity (16% of the outpatients sample), inflammatory bowel disease (31%), hypertension (31%), diabetes mellitus type 1 (22%). † Physical activity level: determined by combining two question, i.e., physical activity at work/school (3 categories) and physical activity at leisure time (3 categories), and assessed in 3 categories: low, moderate and high (i.e., high PA at work/school and high PA at leisure time). ‡ pHDI-10: Pro-Healthy-Diet-Index-10, including 10 questions no.: 23, 25, 31–33, 37, 38, 40, 42–43, evaluated in 3 categories: low (0–33 points), moderate (34–66 points), high (67–100 points). § tertiles of the diet quality indexes were determined separately for each study group in test and retest of the questionnaire. ¶ Nutrition knowledge level: evaluated based on 25 questions no. 55–79 (with 3 response categories: true, false, unsure) and assessed in 3 categories: insufficient (0–8 points), sufficient (9–16 points), good (17–25 points). ¥ nHDI-14: Non-Healthy-Diet-Index-14, including 14 questions no.: 22, 24, 26–29, 34–36, 44, 46, 51–52, 54, evaluated in 3 categories: low (0–33 points), moderate (34–66 points), high (67–100 points).

## Supplementary Materials

The following are available online at http://www.mdpi.com/2072-6643/10/12/1845/s1, Table S1: List of analysed items from the Dietary Habits and Nutrition Beliefs Questionnaire (KomPAN). Table S2: Internal reliability (Cronbach’s alpha for a total score and after each item exclusion) of diet quality scores and nutrition knowledge score in the Dietary Habits and Nutrition Beliefs Questionnaire (KomPAN).
