# Supplementary material for: Reproducibility of a Questionnaire for Dietary Habits, Lifestyle and Nutrition Knowledge Assessment (KomPAN) in Polish Adolescents and Adults
Source: Nutrients. 2018 Dec 1;10(12):1845. doi: 10.3390/nu10121845 (PMC6315932; doi:10.3390/nu10121845)
Supplement: Supplementary file 1 [file nutrients-10-01845-s001.pdf]

Supplementary materials

Table S1. List of analysed items from the Dietary Habits and Nutrition Beliefs Questionnaire (KomPAN).

Questionnaire section/questionnaire item	No.
**Dietary habits**	
Number of meals a day	7
Regularity of consuming meals	8
Frequency of snacking between meals	9
Snacking – fruit	10.1
Snacking – vegetables	10.2
Snacking – unsweetened dairy beverages and desserts	10.3
Snacking – sweetened dairy beverages and desserts	10.4
Snacking – sweet snacks	10.5
Snacking – savoury snacks	10.6
Snacking – nuts, almonds, seeds	10.7
Type of milk and dairy beverages (by fat content)	11
Type of heat treatment of meat – boiled	12.1
Type of heat treatment of meat – stewed	12.2
Type of heat treatment of meat – grilled	12.3
Type of heat treatment of meat – roasted	12.4
Type of heat treatment of meat – fried	12.5
Type of heat treatment of meat – I don't eat meat	12.6
Type of bread spread	13
Type of frying fat	14
Sweetening hot beverages	15
Adding salt to meals	16
Type of water consumed – I don't drink water	17.1
Type of water consumed – still water	17.2
Type of water consumed – sparkling water	17.3
Type of water consumed – flavoured water	17.4
**Food frequency consumption**	
White bread	22
Wholemeal bread	23
White rice, white pasta, fine-ground groats	24
Buckwheat, oats, wholegrain pasta, other coarse-ground groats	25
Fast foods	26
Fried foods	27
Butter	28
Lard	29
Vegetable oils, margarines, mixes of butter and margarines	30
Milk	31
Fermented milk beverages	32
Fresh cheese curd products	33
Cheese	34
Cold meats, smoked sausages, hot-dogs	35
Red meat	36
White meat	37
Fish	38
Eggs	39
Pulse-based foods	40
Potatoes (excluding chips and crisps)	41
Fruit	42
Vegetables	43
Sweets	44

Questionnaire section/questionnaire item	No.
Instant soups, ready-made soups	45
Tinned meat	46
Tinned vegetables	47
Fruit juices	48
Vegetable juices, fruit and vegetable juices	49
Sweetened hot beverages	50
Sweetened beverages	51
Energy drinks	52
Water	53
Alcoholic beverages	54
**Nutrition beliefs**	
It is enough to eat wholegrains/cereals once a day.	55
Only children and adolescents should drink milk.	56
Fruit and/or vegetables should be consumed with every meal.	57
Consumption of mouldy bread can result in food poisoning caused by Salmonella.	58
High intakes of salt protect from hypertension.	59
Limiting high-fat foods in everyday diet is protective against cardiovascular diseases.	60
Frequent consumption of oily fish contributes to atherosclerosis.	61
Frequent consumption of grilled meats contributes to the onset of cancer.	62
Vegetarian diet increases the risk of anaemia.	63
Bio-yoghurts contain beneficial gut bacteria.	64
Vegetable oils and olive oil contain a high amount of cholesterol.	65
Wholemeal bread have more fibre than white bread.	66
Fruit and vegetables are a source of ‘empty calories’.	67
Butter and fortified margarines have high content of vitamin A and D.	68
Cheese is a better source of calcium than cottage cheese.	69
Offal has high amounts of ‘bad’ cholesterol - LDL.	70
In a healthy diet, complex carbohydrates should be replaced with simple sugars.	71
In a balanced diet, proteins should be the main source of energy.	72
Inadequate intakes of vitamin PP can cause skin inflammation and diarrhoea.	73
Sun exposure increases the synthesis of vitamin D in the human body.	74
Phosphorus is a component of neural tissue.	75
The ratio of calcium to phosphorus in a healthy diet should be 1:1.	76
Consumption of fruit with high content of vitamin C increases bioavailability of iron.	77
Starting cooking vegetables in cold water helps to preserve the nutrients.	78
Sweets and animal fats are particularly high nutrient dense foods.	79
**Lifestyle and personal data**	
Following a diet (currently)	80
Eating out	83
Type of alcohol usually consumed	84
Smoking currently	85
Smoking in the past	86
Sleep time on weekdays	87
Sleep time on weekends	88
Time spend watching TV or using a computer	89
Physical activity at work/school	90
Physical activity at leisure time	91
Self-assessment of health status compared to other people of the same age	92
Self-assessment of nutrition knowledge	93
Self-assessment of diet	94
Self-assessment of diet during weekdays compared to weekend	95

No. – item number in the questionnaire.

Table S2. Internal reliability (Cronbach's alpha for a total score and after each item exclusion) of diet quality scores and nutrition knowledge score in the Dietary Habits and Nutrition Beliefs Questionnaire (KomPAN).

No.	Questionnaire Items	Interviewer-Administered Questionnaire	Self-Administered Questionnaire	
		Healthy People	Healthy People	Outpatients#
	Sample size	299	517	138
	**pHDI-10†**	**0.67**	**0.71**	**0.66**
23	Wholemeal bread	0.65	0.69	0.62
25	Buckwheat, oats, wholegrain pasta or other coarse-ground groats	0.64	0.70	0.62
31	Milk	0.65	0.70	0.70
32	Fermented milk beverages	0.64	0.67	0.59
33	Fresh cheese curd products	0.64	0.68	0.64
37	White meat	0.66	0.71	0.67
38	Fish	0.66	0.71	0.65
40	Pulse-based foods	0.67	0.71	0.65
42	Fruit	0.59	0.67	0.61
43	Vegetables	0.59	0.66	0.59
	**nHDI-14‡**	**0.60**	**0.59**	**0.37**
22	White bread	0.58	0.54	0.32
24	White rice, white pasta, fine-ground groats	0.60	0.59	0.37
26	Fast foods	0.57	0.59	0.36
27	Fried foods	0.56	0.57	0.32
28	Butter	0.60	0.58	0.36
29	Lard	0.57	0.59	0.37
34	Cheese	0.60	0.56	0.37
35	Cold meats, smoked sausages, hot-dogs	0.56	0.54	0.31
36	Red meat	0.57	0.58	0.35
44	Sweets	0.56	0.58	0.37
46	Tinned meat	0.59	0.59	0.36
51	Sweetened beverages	0.56	0.56	0.32
52	Energy drinks	0.57	0.58	0.37
54	Alcoholic beverages	0.59	0.59	0.38
	**Nutrition knowledge score§**	**0.80**	**0.80**	**0.73**
55	It is enough to eat wholegrains/cereals once a day.	0.80	0.80	0.73
56	Only children and adolescents should drink milk.	0.80	0.80	0.73
57	Fruit and/or vegetables should be consumed with every meal.	0.79	0.79	0.72
58	Consumption of mouldy bread can result in food poisoning caused by Salmonella.	0.79	0.80	0.73
59	High intakes of salt protect from hypertension.	0.79	0.79	0.73
60	Limiting high-fat foods in everyday diet is protective against cardiovascular diseases.	0.79	0.79	0.73
61	Frequent consumption of oily fish contributes to atherosclerosis.	0.79	0.79	0.72
62	Frequent consumption of grilled meats contributes to the onset of cancer.	0.79	0.78	0.73
63	Vegetarian diet increases the risk of anaemia.	0.79	0.79	0.73
64	Bio-yoghurts contain beneficial gut bacteria.	0.79	0.79	0.72
65	Vegetable oils and olive oil contain a high amount of cholesterol.	0.80	0.79	0.73
66	Wholemeal bread have more fibre than white bread.	0.80	0.79	0.73

No.	Questionnaire Items	Interviewer-Administered Questionnaire	Self-Administered Questionnaire	
		Healthy People	Healthy People	Outpatients#
67	Fruit and vegetables are a source of 'empty calories'.	0.80	0.79	0.73
68	Butter and fortified margarines have high content of vitamin A and D.	0.79	0.78	0.71
69	Cheese is a better source of calcium than cottage cheese.	0.79	0.78	0.73
70	Offal has high amounts of 'bad' cholesterol - LDL.	0.79	0.78	0.72
71	In a healthy diet, complex carbohydrates should be replaced with simple sugars.	0.79	0.79	0.73
72	In a balanced diet, proteins should be the main source of energy.	0.80	0.79	0.73
73	Inadequate intakes of vitamin PP can cause skin inflammation and diarrhoea.	0.79	0.78	0.71
74	Sun exposure increases the synthesis of vitamin D in the human body.	0.79	0.79	0.72
75	Phosphorus is a component of neural tissue.	0.79	0.78	0.71
76	The ratio of calcium to phosphorus in a healthy diet should be 1:1.	0.79	0.79	0.73
77	Consumption of fruit with high content of vitamin C increases bioavailability of iron.	0.79	0.79	0.72
78	Starting cooking vegetables in cold water helps to preserve the nutrients.	0.80	0.79	0.73
79	Sweets and animal fats are particularly high nutrient dense foods.	0.79	0.79	0.73

No. – item number in the questionnaire. #overweight/obesity (16% of the outpatients sample), inflammatory bowel disease (31%), hypertension (31%), diabetes mellitus type 1 (22%). †pHDI-10 – Pro-Healthy-Diet-Index-10 including 10 questions no.: 23, 25, 31-33, 37, 38, 40, 42-43 (the total score range: 0-100). ‡nHDI-14 – Non-Healthy-Diet-Index-14 including 14 questions no.: 22, 24, 26-29, 34-36, 44, 46, 51-52, 54 (the total score range: 0-100). §Nutrition knowledge score – evaluated based on 25 questions no. 55-79, with 3 response categories: true, false, unsure (the total score range: 0-25).
